# Modulatory effects of α7 nAChRs on the immune system and its relevance for CNS disorders

**DOI:** 10.1007/s00018-016-2175-4

**Published:** 2016-03-15

**Authors:** Hans O. Kalkman, Dominik Feuerbach

**Affiliations:** Neuroscience Research, NIBR, Fabrikstrasse 22-3.001.02, 4002 Basel, Switzerland; Gänsbühlgartenweg 7, 4132 Muttenz, Switzerland

**Keywords:** Dyskinesia, Autism, Suicide, Lithium, GSK3, CREB, Nrf2

## Abstract

The clinical development of selective alpha-7 nicotinic acetylcholine receptor (α7 nAChR) agonists has hitherto been focused on disorders characterized by cognitive deficits (e.g., Alzheimer’s disease, schizophrenia). However, α7 nAChRs are also widely expressed by cells of the immune system and by cells with a secondary role in pathogen defense. Activation of α7 nAChRs leads to an anti-inflammatory effect. Since sterile inflammation is a frequently observed phenomenon in both psychiatric disorders (e.g., schizophrenia, melancholic and bipolar depression) and neurological disorders (e.g., Alzheimer’s disease, Parkinson’s disease, and multiple sclerosis), α7 nAChR agonists might show beneficial effects in these central nervous system disorders. In the current review, we summarize information on receptor expression, the intracellular signaling pathways they modulate and reasons for receptor dysfunction. Information from tobacco smoking, vagus nerve stimulation, and cholinesterase inhibition is used to evaluate the therapeutic potential of selective α7 nAChR agonists in these inflammation-related disorders.

## Introduction

Alpha-7 nicotinic acetylcholine receptors (α7 nAChRs) are expressed in the central nervous system (CNS) and are thought to play a role in a wide variety of psychiatric and neurological disorders. Peripheral and CNS immune cells strongly express α7 nAChRs; activation of α7 nAChRs on these cells has been shown to suppress inflammatory processes. Since inflammation is involved in several psychiatric disorders, as well as in basically all neurological disorders, specific α7 nAChR agonists could display therapeutic effects. In the current review, we summarize information on receptor expression, the intracellular signaling pathways modulated by these receptors, reasons for receptor dysfunction, clinical evidence for altered α7 nAChRs, and we conclude with a discussion about potential indications for selective α7 nAChRs agonists.

## Expression of α7 nAChR

Distinct from most nicotinic acetylcholine receptors (nAChR), the α7-subtype mainly forms homomeric, rather than heteromeric pentamers. In the central nervous system, including the human brain, such nicotinic-α7 homomeric pentamers are expressed by pyramidal and interneurons [1–3]. Apart from neurons, immature (doublecortin positive) granule cells [4], astrocytes [5–7], and microglia cells [8–13] also express the α7 nAChR. Finally, NG2-positive cells (these are oligodendrocyte precursors) also express α7 nAChRs [5, 14]. The dogma that α7 nAChRs exclusively assemble as homomeric pentamers was recently overturned by the discovery that α7 subunits also form heteromeric pentameric ion channels with β2 subunits [15]. Such heteromeric α7β2 channels were found on cholinergic projection neurons in mouse and human basal forebrain [15, 16]. Concerning function, activation of α7 nAChRs results in strong calcium and sodium influxes, which in the case of a presynaptic location facilitates neurotransmitter release [17, 18]. A postsynaptic localization on parvalbumin-positive GABA neurons suggests a role in synchronized oscillatory output of pyramidal neurons.

Outside the brain, the receptor is expressed on several cell types of the immune system. This includes monocytes [19–21], dendritic cells [22], macrophages [23–26], T-cells [27, 28], and B-cells [29, 30]. The receptor has also been identified on additional cell types known to play a role in the host’s defense against pathogenic organisms. Examples are the microvascular endothelium [31], keratinocytes [32, 33], placenta [34], bronchial epithelial cells [35], platelets [36], adipocytes [37], and synoviocytes [38]. During wound healing, expression of α7 nAChRs is transiently observed on fibrocytes and myofibroblasts in the wound zones [39]. Completing this list, α7 nAChRs were detected in mouse testes, in mouse and human sperm (where they modify sperm motility [40]), in rat superior cervical ganglia [41], and in group-IV muscle afferent neurons [42].

Not unexpectedly, numerous cell lines *endogenously* express α7 nAChR too. This includes rat PC12-cells [43], human SH-SY5Y neuroblastoma cells [44, 45], mouse RAW264.7 [46, 47], the human leukemic T-cell line MOLT3 [48], the human monocyte U937 cell line [20, 49], and immortalized human T-lymphocyte ‘Jurkat’ cells [27]. *Recombinant* expression has also been achieved in cell lines that endogenously express the chaperones required for α7 nAChR expression, e.g., SH-EP1 cells [50], SH-SY5Y [51], or GH3 cells [52].

With respect to the above-listed localizations of α7 nAChR, a word of caution is needed, since some expression studies are confounded by the use of tools that also detect a distinct, duplicated α7-like protein ‘dupα7’ [26, 53], by the use of antibodies which recognize cross-reacting epitopes [54] or by the use of the non-selective radioligand MLA (besides α7 nAChRs this compound also binds to nicotinic α3/α6β2β3* receptors, see [55]).

In addition to expression on the cell surface, an intracellular localization of α7 nAChRs has been observed in brain mitochondria [56]. In this organelle, the α7 nAChR may assemble with β2 subunits where it presumably influences pore formation and cytochrome-c release [57].

## Intracellular signaling pathways

**Fig. 1:**
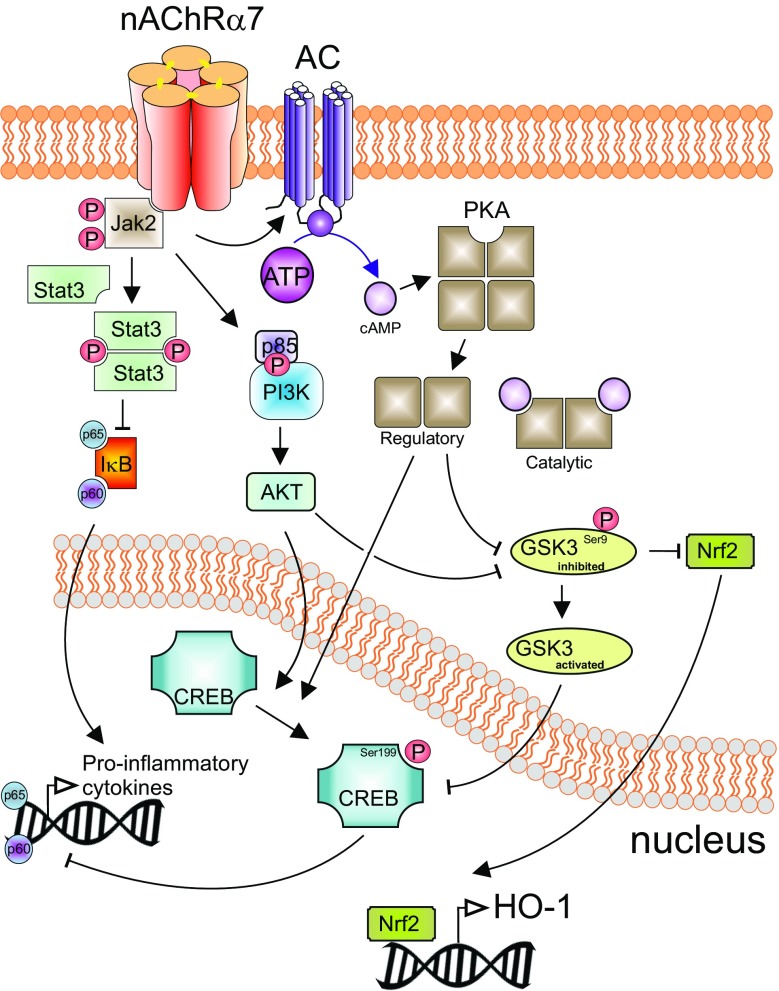
Schematic anti-inflammatory signaling pathways activated by nAChR α7. Stimulation of nAChR α7 activates Jak2 leading to inhibition of NFκB and GSK3 but also to CREB activation. A separate signaling cascade involves activation of PKA and AKT enabling the nuclear translocation of Nrf2 (NFE2L2), which drives expression of HMOX1 (HO-1). This pathway elicits potent anti-inflammatory and neuroprotective effects

In mouse hippocampal neurons, α7 nAChRs are characterized by rapid activation and desensitization. The fractional calcium current (*P*_f_) has been assessed and found to be 6.1 [58]. This indicates that in neurons α7 nAChRs have a special role in the modulation of intracellular calcium, enabling substantial calcium entry at resting or hyperpolarized membrane potential, which is different compared to other nAChRs, but similar to NMDA receptors at depolarized membrane potential. However, thus far, it is not known if this also holds true for α7 nAChRs in other cells (e.g., immune cells). The intracellular pathways following α7 nAChR activation in non-neuronal cells involve calcium influx through the channel pore, which then triggers calcium-induced calcium release from ryanodine- and IP3-dependent stores [10, 28, 59]. Further long-lasting intracellular pathways involve activation of intracellular phosphatases and kinases, which trigger signaling events *independent* of an ion flux (which implies that the α7 nAChR acts as metabotropic receptor) [60]. For instance, activation of α7 nAChRs leads to stimulation of adenylate cyclase-1 and thus to increases in cAMP levels [61]. This in turn stimulates protein kinase A (PKA), which may result in further signaling events such as CREB activation [61] and GSK3 inhibition [62]. Activation of the α7 nAChR on non-neuronal cells inhibited TLR3-, TLR4- or TLR9-induced transcription and release of inflammatory cytokines [9, 19, 21, 34, 47]. One of the intracellular signaling cascades described in this context is a pathway that involves JAK2-mediated tyrosine-phosphorylation of the p85 subunit of PI3 K, activation of Akt and CREB, and subsequent inhibition of (or competition with) NFκB [20, 25, 49, 63] (see Fig. 1). Egea and colleagues emphasize that this pathway furthermore leads to activation of the transcription factor Nrf2, which is important for transcription of numerous anti-oxidative proteins and for the induction of an anti-inflammatory phenotype of microglia cells [64]. Alternatively, downstream signaling towards NFκB may involve JAK2 activation of STAT3 [65–68] (see Fig. 1). Finally, activation of α7 nAChRs can result in inhibition of p38 MAP-kinase [8, 10, 19]. A functional consequence of this latter pathway is inhibition of the *release* of inflammatory mediators like TNFα and HMGB1 [8, 10, 19].

## The anti-inflammatory activity of α7 nAChR stimulation

As early as 1998, Sugano et al. [49] described that nicotine displayed an anti-inflammatory activity involving inhibition of NFκB-signaling. Following this observation, it was shown that the receptor responsible for this response was the α7 nAChR subtype [20, 23, 24, 69, 70]. Moreover, it was shown that the anti-inflammatory effect of electrical stimulation of the vagus nerve was also mediated by the α7 nAChR [23, 24, 69]. Notably, after splenectomy the beneficial effects of vagus nerve stimulation were lost [71], but some controversy still exists about the exact localization of the α7 nAChRs involved in the response to vagus nerve stimulation. The vagus nerve is supposed to activate the celiac ganglion, which is the origin of the adrenergic splenic nerve. According to one scenario, the splenic nerve releases noradrenaline onto *memory* T-cells (CD4^+^ CD44^high^, CD62L^low^), resulting in synthesis and release of acetylcholine that activates α7 nAChRs on spleen-macrophages [69, 72, 73]. The alternative proposal assumes that the α7 nAChRs are localized postsynaptically in the celiac ganglion. This view is supported by data showing that postganglionic stimulation of the splenic nerve still results in an anti-inflammatory response, even in mice with a genetic deletion of the α7 nAChR [74]. However, in a critical review Martelli [75] proposes that efferent vagus nerve stimulation achieves its anti-inflammatory effect via a non-neuronal (humoral) pathway. It is evident that this is an area that is still very much in development. Interestingly, activation of glucocorticoid receptors increases the expression of α7 nAChRs [76]. This implies that the anti-inflammatory activity of glucocorticoids may include a nicotinergic mechanism. Overall, given the expression pattern, the cellular signaling cascades, and information from vagus nerve stimulation, the data strongly indicate that activation of the α7 nAChR suppresses the responsiveness of the immune system.

Surprisingly, whereas the activity of positive allosteric modulators (PAMs) in animal models of learning/memory or on evoked potentials is well documented (see [77] for a recent review), there is a paucity of reports on their anti-inflammatory activity. In this context, PNU-120596 has been shown to attenuate TNFα and IL-6 in a rodent model of inflammatory pain. These findings have been recently corroborated with a different molecule (“PAM-2,” [78]). Along the same lines several reports have shown that α7 nAChR PAMs reduce brain injury and improve neurological function after focal cerebral ischemia in rats [79–81].

Notably, the anti-inflammatory response to α7 nAChR stimulation also occurs in the brain. Thus, activation of α7 nAChRs is known to alter the phenotype of both macrophages and microglia from an M1-like to an M2-like phenotype [11, 19, 82, 83]. Consequently, any dysfunction in the α7 nAChR and its signaling processes could tip the balance towards more inflammation.

## Reasons for dysfunction of the α7 nAChR

Dysfunction of α7 nAChRs may have a variety of causes. For adequate membrane insertion, the α7 nAChR has to be assembled first as a pentamer and is thereafter shuttled to the plasma membrane. This process requires several chaperone molecules such as RIC3, SLURP1, Lynx1, EPHB2, or PICK1, and evidently, their dysfunction could affect receptor level and function [1, 33, 48, 84–88]. In addition, transcription of the α7 nAChR can be diminished by heterozygotic or homozygotic 15q13.3 microdeletions [89], by methylation of the promotor [90] or by MeCP2 (methyl CpG binding protein 2) dysfunction [91].

Moreover, activity of the α7 nAChR is modified by phosphorylation. An as yet undefined tyrosine-phosphorylated protein was shown to inhibit the functional activity of the α7 nAChR, whereas tyrosine kinase inhibition by genistein enhanced surface expression of the α7 nAChR [35, 37, 84, 92] or its function [51]. Serine-phosphorylation of the α7 nAChR can influence function too. It has been reported that activation of D1/D5 dopamine receptors attenuated α7 nAChR currents via PKA-mediated phosphorylation of the serine residue 365 in the M3–M4 cytoplasmic loop of the channel [93]. Other possibilities have been proposed as well. For instance, diminished signaling of α7 nAChR might result from increased levels of the purported endogenous α7 nAChR inhibitor, kynurenic acid [94], though conflicting data have been reported too [95]. Also cholinergic input may be dysfunctional [69], or the signaling pathway downstream of the receptor may be altered [27, 66, 92]. Furthermore, dysfunction of the α7 nAChR could result from the presence of the CHRFAM7-gene. This gene encodes a dominant negative variant of the α7 nAChR [26, 96] and is unique for humans [26, 97]. Transcripts, often described as ‘dupα7’, have been found in the promyelocytic leukemia cell line HL-60 [26], monocyte cell lines (THP1, U937, and Mono-Mac6 [98]), and neuroblastoma cell lines (SH-SY5Y, IMR32 [98]). High levels of dupα7 transcripts have also been described in native immune cells such as peripheral blood monocytic cells, lymphocytes and synoviocytes, and in lower amounts also in human brain tissue [7, 26, 53].

## Rapid metabolism of acetylcholine and receptor desensitization

Cholinergic innervation in the brain arises mainly from two sources. Cholinergic neurons from the medial septal nucleus innervate the hippocampus [99], whereas cholinergic nerves from the basal forebrain (including the nucleus basalis Meynert) project to the cortex, amygdala, caudate nucleus, putamen, and thalamus [100]. Acetylcholine locally released by cholinergic neurons is assumed to cross the synaptic cleft despite effective catabolism by cholinesterases [101]. However, it is less clear whether volume transmission to extrasynaptic neuronal sites is sufficiently high to lead to relevant α7 nAChR stimulation. Outside the brain, high levels of cholinesterases are found in the blood and therefore it has been questioned whether α7 nAChRs on, for example, circulating immune cells will be exposed to levels of acetylcholine that are sufficient for activation [102]. Further doubts about the physiological relevance of α7 nAChRs relate to their rapid desensitization [101, 103, 104]. However in the latter case, there are two counter-arguments. In the first place, α7 nAChRs not only rapidly desensitize, they also quickly recover [105]. The second argument is that intracellular effects are presumably more sustained and therefore could outlast receptor desensitization. Vijayaraghavan et al. [6] provide yet one more piece of information, which, in addition, addresses the issue of volume transmission. The authors found that *extracellular* levels of choline-acetyl transferase (ChAT) are actively regulated. This means that, despite ongoing extracellular cholinesterase-activity, extracellular acetylcholine is permanently resynthesized. As a consequence, acetylcholine may act over long distances from its site of release. Importantly, acetylcholine is not only synthesized and released by neurons but also by several non-neural cells. Frequently these cells express both ChAT and α7 nAChRs, and as a result, an intrinsic paracrine loop is formed [102]. Examples are bronchial epithelial cells [35], lymphocytes [6, 28], human neuronal stem cells [6], and astrocytes [6, 106]. As a last argument in favor of a physiological relevance of extrasynaptic α7 nAChRs, it should be mentioned that these receptors are also activated by choline, the precursor and hydrolytic split product of acetylcholine [46, 67, 81, 107]. In summary, extrasynaptic α7 nAChRs will be readily activated under physiological conditions.

## Diseases in which treatment with α7 nAChR agonists could be useful

Since α7 nAChRs are expressed on interneurons, presynaptically on glutamatergic neurons, and on neuronal progenitor cells, the logical prediction was that α7 nAChR agonists could provide a beneficial influence on cognitive function. Consequently, the clinical testing of α7 nAChR agonists has focused on disorders with profound cognitive dysfunction (i.e., schizophrenia, Alzheimer’s disease). The fact that nicotinic α7 receptors are also strongly expressed by cells of the immune system, including those of the brain innate immune system, has often been (but not always, [64, 108]) overlooked. With this in mind, in the following sections we will reflect on the therapeutic use of selective α7 nAChR agonists in psychiatric and neurological disorders.

## Depression

**Fig. 2:**
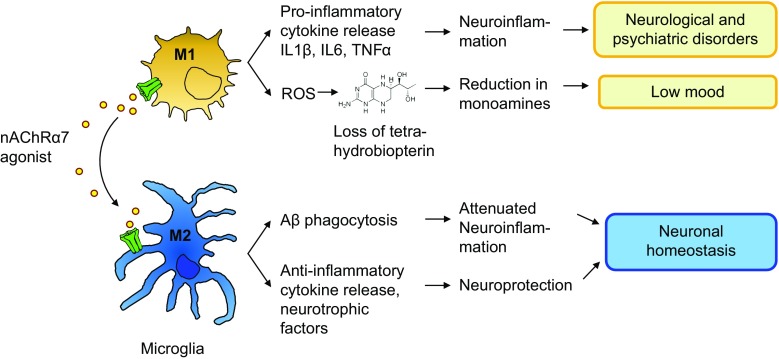
Microglia cells play a role in eradication of invading microorganisms and in removal of debris. During these processes, the cells adapt specialized phenotypes (M1 and M2-like, respectively). During the respiratory burst, labile oxygen products are formed that oxidize microbial proteins and nucleic acids, but also oxidize the essential cofactor for monoamine synthesis, tetrahydrobiopterin. Sterile neuroinflammation is a common observation in neurological and psychiatric disorders. Acetylcholine, acting via α7 nAChRs, promotes M2-polarization. As such, it reduces neuroinflammation, while promoting phagocytosis. M2-polarized microglia cells not only produce neurotrophins and anti-inflammatory cytokines, they also effectively phagocytose and catabolize Aβ. α7 nAChR agonists are expected to improve neurological and psychiatric disorders via inhibition of neuroinflammation, to restore tetrahydrobiopterin levels (improve mood symptoms) and to provide neuroprotection

Inflammation and exposure to stress are generally recognized as strong proximal factors for the development of depression symptoms [109]. Many kinds of stress, but in particular those involving threat, loss, entrapment, and humiliation, lead to activation of the immune system [109–112]. This has been conceptualized as an anticipatory response by the innate immune system to prepare for physical injury [113]. Phagocytic cells such as macrophages and microglia are essential components of the innate immune system and contribute to the host’s defense against invading microorganisms. Notably, phagocytes from patients with depression are hyper-responsive [114–116]. Upon activation, these cells produce cytotoxic compounds like nitric oxide and oxygen radicals [117, 118]. These reactive oxygen radicals formed during the ‘respiratory burst’ may irreversibly oxidize tetrahydrobiopterin [119]. Since tetrahydrobiopterin is an essential cofactor for the production of dopamine, noradrenaline, and serotonin, the heightened activity of microglial cells may ultimately lead to a reduction in central monoamine levels (see Fig. 2). This could be the underlying cause of low mood and anhedonia in depression (Kalkman and Feuerbach, in preparation). As outlined above, α7 nAChRs are expressed both on macrophages and microglia, and their activation leads to anti-inflammatory effects. In particular, activation of α7 nAChRs might lead to a shift in microglia phenotype from M1-like (activated for anti-microbial activity) to M2-like (resolution, removal of debris) [10, 11, 13, 83, 120], while a similar process has been described in the periphery. Stimulation of α7 nAChRs attenuates macrophage responsiveness and diminishes release of cytokines [23, 46, 47, 66] and therefore will limit the negative impact of inflammation on tetrahydrobiopterin metabolism.

Activation of α7 nAChRs in the brain has been shown to activate protein kinase A (PKA) via a mechanism involving calcium-dependent activation of adenylatecyclase-1 and, consequently, an increase in cAMP levels [61]. PKA is one of the kinases that increase Ser9-phosphorylation of GKS3β, which results in inhibition of the kinase activity. PKA also fosters Ser133-phosphorylation of the transcription factor CREB. Ser133-P-CREB is a substrate for GSK3β, which subsequently results in rapid catabolism of CREB and thus termination of CREB signaling. CREB competes with NFκB for binding to CREB-binding protein (CBP), and therefore limits the inflammatory NFκB signal. Activation of α7 nAChRs in the brain may thus lead to an anti-inflammatory activity. Increased PKA activity, increased Ser9-phosphorylation of GSK3β, and increased Ser133-phosphorylation of CREB have all been observed in mouse brain after chronic treatment with the α7 nAChR agonist A582941 [62]. Notably, Ser9-phosphorylation of GSK3β following A582941 treatment was absent in mice with a genetic deletion of the α7 nAChR [121].

Vagus nerve stimulation, which reduces inflammation [23, 66, 69, 122], has been approved for treatment of drug-resistant depression in humans [123]. In long-term naturalistic studies, significant improvements and increasing remission rates were noted in patients with refractory depression [124–126]. Oxytocin (reported to display antidepressant activity [127]) increases the excitability of central vagal neurons in rats [128] and inhibits LPS-induced release of inflammatory cytokines in healthy human subjects [129]. The antidepressant activity of oxytocin could thus be a consequence of stimulation of the cholinergic anti-inflammatory pathway. In conclusion, there is accumulating evidence that α7 nAChR agonists could possess antidepressant activity, although none of the compounds that were in clinical development has been tested in this indication.

## Schizophrenia

Symptoms of schizophrenia are commonly divided into three domains, namely positive symptoms (delusions, hallucinations), negative symptoms (social withdrawal, anhedonia), and cognitive deficits (learning and memory deficits, alogia). A history of maternal and prenatal infections, prior hospitalization for severe infection, and autoimmune comorbidity represent major risk factors for schizophrenia [130]. These conditions, which are linked to the elevation of pro-inflammatory cytokines has led to the formulation of the “prenatal cytokine hypothesis” [131]. This hypothesis proposes that early alterations in the peripheral and central innate immune system disrupt normal development and maturation of neuronal systems during the juvenile and early adult stages of life, affecting processes such as myelination, synaptic pruning, and neuronal modeling [131]. Results from genetic studies are consistent with such a process, as confirmed genetic risk factors for schizophrenia include mutations in genes involved in immune function (e.g., HLA-C and HLA-DRA) and synaptic pruning [132, 133]. The first episode of psychosis is often associated with microglia activation [130, 134, 135], elevated levels of pro-inflammatory cytokines in the CSF [136–138], and a significant loss in white matter volume (reviewed in [136]). It is assumed that cytokines, chemokines, prostaglandins, and reactive oxygen products released by activated microglia generate a toxic milieu for oligodendrocytes (leading to white matter loss) and neurites (gray matter loss) [130, 136, 139, 140]. The severity of negative symptoms correlates with the diminution of white matter [130].

The impetus to develop α7 nAChR agonists was based on two sets of clinical information. First, the early literature reported extremely high values for the prevalence of smoking in schizophrenia patients (see [141]). This information was supplemented by data from radioligand binding experiments investigating brain tissue of deceased schizophrenia patients (for summary see [100]). Using [^125^I]-α-bungarotoxin (α-btx) as radioligand, a *reduction* in labeling of the α7 nAChR was found in the dentate gyrus [142], the reticular nucleus of the thalamus [143], the frontal cortex [144], and the cingulate cortex [1]. A vast body of genetic literature supports the contention that the functionality of the α7 nAChR in schizophrenia is diminished. CHRNA7, the gene encoding the α7 nAChR, is located on 15q14, a chromosomal area that is linked to genetic transmission of schizophrenia [145]. This area is also subject to deletion copy number variations (CNVs,) [146, 147]. Decreases in α-btx binding observed in schizophrenia patients could be due to an increased expression and insertion of dupα7, since heteromeric dupα7/α7 nAChRs do not bind this radioligand [84, 148]. Notably, decreased α-btx binding is unlikely to be explained by differences in smoking behavior [1]. Polymorphisms in the CHRNA7 promoter that decrease gene-transcription are also associated with schizophrenia [149]. A 2 bp deletion allele in CHRFAM7A is frequent in Caucasians (42 %) and less in African-Americans (14 %) [150]. This 2 bp deletion form of CHRFAM7A is an even stronger inhibitor of α7 nAChR than wild-type CHRFAM7A and consistent with this, several studies support an association of the 2 bp deletion in the CHRFAM7A gene with schizophrenia and bipolar disorder (summarized by Sinkus et al. [150]). Finally, CNVs occur in the CHRNA7 and in CHRFAM7A, and deletions were strongly associated with schizophrenia. Interestingly, this was especially the case when the CHRNA7 was deleted, while CHRFAM7A was present [150].

The pathophysiological consequence of the diminished functionality was mainly sought in the cognitive domain. The prediction that α7 nAChR agonists would improve cognition in schizophrenia was tested clinically with a series of development compounds (for a recent reviews, see [100, 151, 152]). Since the nicotine α7 receptor and the 5HT3 receptor are phylogenetically very similar, several developmental α7 nAChR agonists lack selectivity and block the 5HT3 receptor concomitantly [108]. Encenicline (EVP6124), which is a mixed α7 nAChR agonist/5HT3 antagonist, showed significant clinical improvement on PANSS cognitive impairment domain and also for the PANSS negative scale [153]. Another mixed α7 nAChR agonist and 5HT3 antagonist tropisetron significantly reduced PANSS total and the negative symptom subscale with increasing treatment time [154]. Further, a mixed α7 agonist/5HT3 antagonist RG3487 (also known as MEM3454), was reported to improve negative symptoms and depression ratings [155]. It remains unclear if the beneficial effect on negative symptom ratings is due to α7 nAChR activation or to 5HT3 blockade, as both a selective α7 nAChR agonist (TC-5619) and a selective 5HT3 antagonist (ondansetron) improved negative symptoms [156, 157]. DMXB-A (GTS-21) [158], a α7 nAChR agonist with additional inhibitory activity at α4β2 nAChRs [159], improved alogia and anhedonia ratings in the scale for assessment of negative symptoms. Remarkably, α7 nAChR-positive allosteric modulators (galantamine, galantamine plus choline, and JNJ39393406) were completely inactive [100]. Taken together, these data show that α7 nAChR activation did not improve positive symptoms, whereas a beneficial effect against negative symptoms was observed repeatedly. The clinical evidence that α7 nAChR activation leads to improvement of cognitive dysfunction in schizophrenia still remains somewhat equivocal [100, 160], although the data for encenicline are encouraging [153, 161]. It is noteworthy that the clinical development of several mixed nAChR α7 agonist/5HT3 antagonist compounds (e.g., RG3487, tropisetron) in schizophrenia has been halted [152]. This may be related to side effects associated with 5HT3 receptor blockade (e.g., constipation, arrhythmias) (see [108, 162, 163]). Currently, three α7 nAChR agonists remain in clinical development (AQW051, encenicline, and GTS-21) [152].

The observation that nAChR α7 agonists improve negative symptoms is remarkable. It can be speculated that stimulation of nAChRs α7 counteracts microglia activation, such that white and gray matters are exposed less to the toxic microglia products. In this respect it is noteworthy that minocycline, a compound which suppresses microglia activation, also specifically improved negative symptoms (for review see [164]). Negative symptoms, like symptoms of melancholic depression, might reflect distinct behavioral consequences of central inflammation. However, it could be that negative symptoms are not distinct and in fact reflect aspects of melancholic depression, or that rating scales for negative symptoms do not discriminate between depression symptoms and negative symptoms [155]. Since microglia activation occurs early in the disease process (i.e., during, or even before, the first period of psychosis), early intervention with compounds that limit microglia activation (nAChR α7 agonists, minocycline) might be appropriate.

## Bipolar disorder

The cardinal features of bipolar disorder are recurrent episodes of depression and hypomania, whereas the latter includes euphoric or irritable mood, mental and behavioral over-activity, as well as decreased need for sleep and involvement in risky activities [165]. As for major depressive disorder, diseases and habits that are associated with peripheral inflammation (diabetes, obesity, cardiovascular disease, smoking, and alcohol abuse) are recognized risk factors for bipolar disorder (reviewed in [166–168]). And in further similarity to major depression, childhood trauma is a strong predictor for appearance of bipolar disorder later in life [169]. Increases in serum levels of inflammation markers have been observed during manic, depressed and even euthymic phases [170–173]. Bipolar disorder shares genetic risk loci with schizophrenia and with major depression [171, 174]. There is evidence for microglia activation in the brain of bipolar disorder patients: first, higher levels of IL1β, IL1R, MYD88, and iNOS have been found in a post-mortem study in the frontal cortex [175]. Second, microglia-derived inflammation markers MCP1 and YKL40 were increased in CSF [176], and third, microglia activation was detected by PK11195 imaging [177]. The inflammatory milieu in the brain is probably responsible for atrophy, volumetric changes, cognitive decline, and symptom worsening [167, 175]. The same genetic polymorphisms, copy number variations and alteration in the pseudo-gene CHRFAM7A, which are presumed to diminish the functionality of the nAChR α7 receptor in schizophrenia, have also been detected in bipolar disorder [161, 178].

The neurodevelopmental consequences of diminished α7 nAChR signaling may be inferred from studying CHRNA7 knockout mice. It has been reported that nicotine administration to these animals led to a longer period of elevated extracellular dopamine levels in the nucleus accumbens than in control mice [179]. Excessive dopamine signaling via D2 receptors in the striatum causes an activation of GSK3β via a multiprotein complex involving the D2 receptor, β-arrestin, Akt, GSK3β, and PP2A [180, 181], while active GSK3β results in diminished long-term potentiation (LTP) [182–184]. Unexpected rewarding outcomes result in dopamine release in the striatum, whereas unpredicted negative outcomes result in a strong reduction in dopamine output [185]. Such a dopamine “dip” improves avoidance learning (via D2 receptor hypo-stimulation) by decreasing GSK3β activity, and thus promoting LTP. In contrast, hyper-stimulation of D2 receptors, and activation of GSK3β, both result in a diminished learning from incorrect reward predictions. If we assume that the α7-p85-Akt-GSK3β pathway is activated in striatal neurons (as it is in immune cells), dysfunction of the α7 nAChR would result in reduced inhibitory GSK3β-phosphorylation, in poorer learning from unrewarding conditions, and eventually in more (i.e., less suppressed) risk-taking behavior. Taken together, dysfunction of the α7 nAChR would increase both depression-risk and hypomania symptoms. The prediction that selective α7 nAChR agonists might counteract these symptoms remains to be tested.

## Autism spectrum disorder

Autism is a general term for a group of neurodevelopmental disorders characterized by difficulties in social interaction, verbal and non-verbal communication, and repetitive behaviors. Several distinct subtypes have been identified, and these are often combined under the umbrella diagnosis “autism spectrum disorder” (ASD). Certain genetic copy number variations, which can be either inherited or occur de novo, exert a profound effect on brain development and lead to syndromes that fit the ASD diagnosis. The 15q13.3 microdeletion syndrome is such an example. Gillentine and Schaaf [147] collected all published cases of heterozygous 15q13.3 deletions and calculated that nearly half of them displayed cognitive deficits, while seizures and symptoms of ASD were noted in 26 and 21 % of these cases, respectively. It is not clarified by which mechanism heterozygosity for the α7 nAChR could lead to ASD. It is known, however, that the α7 nAChR is essential for the formation of NMDA synapses [86], and that the absence of functional α7 nAChRs results in perturbation of NMDA neurotransmission at glutamatergic synapses [186]. The EphB2 receptor is involved in the process by which α7 nAChRs enhance formation of NMDA synapses [86, 187] and, interestingly, also dysfunction of the EphB2-receptor has been recognized as a risk factor for autism spectrum disorder [188].

Rett syndrome is another distinct genetic form of autism. In the majority of cases, Rett syndrome is caused by a mutation in the MeCP2 gene; the functional consequence of this mutation is a severe reduction in the expression of α7 nAChRs [91]. Yasui and colleagues have therefore proposed to use α7 nAChR agonists for the treatment of Rett syndrome.

Although the pathology seen after partial deletion or dysfunction of the α7 nAChR provides support for its physiological role, it also creates a dilemma for pharmacotherapy with α7 agonists. Such compounds can only work when the receptor is present and functional. A study by Le Pichon and colleagues exemplifies this [189]. The authors created lymphoblastoid cell lines from a patient with a *homozygous* 15q13.3 microdeletion, his heterozygous parents, and from several further family members and age-matched controls. The lymphoblast cells were treated with lipopolysaccharide, which provoked the expected increase in TNFα release. TNFα release was suppressed by nicotine (which in turn could be blocked by the α7 nAChR antagonist α-btx) in all control and hemizygous deletion cell lines, but notably *not* in lymphoblasts from the index patient [189]. Patients with a strongly dysfunctional or absent α7 nAChR will not benefit from a α7 nAChRs agonist. It is reasonable to assume that cases of moderate hypoactivity of the α7 nAChR will exist among the wide spectrum of autism disorders, but the challenge will be to identify a biomarker to enable their selection. The current clinical evidence for a therapeutic effect of an increase in cholinergic signaling in ASD is quite limited. It is based on a few open label studies, case reports and a single small-size double-blind augmentation study with a cholinesterase-inhibitor [190].

In a subset of autism patients, there is evidence for an increase in allergic inflammation [191] and for mast cell activation [192]. These observations hint to an increased T_H_2 polarization (perhaps owing to a helminth infection). An infection with a helminth would skew the differentiation of helper T-cell towards the T_H_2 phenotype. IL4 and IL5, cytokines produced by T_H_2 cells [193], promote macrophage/microglia M2A polarization and mast cell activation [194–196]. Indeed, Gupta et al. [197], recently reported that the expression pattern of microglia genes in autism cases was characteristic for an increased M2A phenotype. Treatment with α7-agonists is expected to further increase M2 polarization, and thus could accentuate the autism-phenotype. According to this line of reasoning, the treatment with α7 nAChR agonists might be contra-indicated in at least some forms of autism spectrum disorder.

## Multiple sclerosis

Neuroinflammation represents a key aspect of the neurological disorders that will be discussed in this and following sections. Multiple sclerosis (MS) is an autoimmune disease that affects axonal nerve transmission in peripheral, lumbal, and central nerves, which as first symptoms often results in motor and sensory problems. Depression is a common comorbidity [198]. The precise sequence of events that leads to MS in patients is not known [199], but activation of lymphocytes, macrophages, dendritic cells, and microglia are known to occur early in the disease [200]. α7 nAChR activation can lead to inhibition of lymphocyte proliferation [28, 201] and to inhibition of macrophage and microglia activation [8, 11, 24, 25, 46]. Stimulation of α7 nAChR on endothelial cells furthermore limits the extravasation of leukocytes during inflammation [31], although it is not known if this is also true for the blood–brain barrier. Together, these findings suggest that α7 nAChR agonists might be therapeutically useful in multiple sclerosis. Indeed, nicotine was shown to inhibit experimental MS in rodents [83, 201], but to our knowledge, selective α7 nAChR agonists remain to be investigated in human MS.

## Parkinson’s disease

The motor symptoms of Parkinson’s disease (PD) are ascribed to degeneration of dopaminergic neurons in the *substantia nigra pars compacta*. Other important symptoms are anxiety and depression, as well as memory loss, confusion, and dementia. In this respect, it is noteworthy that not only dopamine neurons perish but also the cholinergic system. Degeneration of the basal forebrain cholinergic system occurs early in the disease process and precedes the dementia symptoms [202, 203]. In fact, in PD-dementia the levels of cerebral ChAT are reduced to levels below those seen in Alzheimer’s disease [204], whereas cholinesterase inhibition improves dementia in Parkinson’s disease [205].

A large number of epidemiological studies (for a recent summary see [120]) have consistently shown that smoking is associated with a lower incidence of PD. Data from preclinical models of Parkinson’s disease indicate that nicotine and selective α7 nAChR agonists reduce microglia activation and neuroinflammation, and prevent *nigro*-*striatal* dopamine-neuronal loss [206, 207]. α7 nAChRs expressed on astrocytes may also contribute to the neuroprotective effect, since activation of these receptors suppressed astroglial apoptosis induced by oxidative stress and preserved neurotrophic factor supply by glial cells [208]. An alternative suggestion is that nicotine reduces PD symptoms owing to its stimulant effect on *nigro*-*striatal* dopamine release. This nicotine-induced dopamine release is mediated primarily via α6β2-containing receptors localized on dopaminergic neurons. These neurons are particularly vulnerable to damage and in models of *nigro*-*striatal* damage, the α6β2-receptor numbers are significantly reduced [209]. For this reason, it is rather unlikely that the indirect dopamine release induced by nicotine acting on α6β2 receptors plays a significant role in the positive effect of smoking in PD. Post-mortem studies of brains from patients with Parkinson’s disease provide convincing evidence for neuroinflammation in the *pars compacta*, with increases in neurotoxic cytokines, microglia activation, and lymphocyte infiltration (reviewed by Hirsch and Hunot [210]).

Depression occurs in about 35 % of PD-patients [211] and might develop in the premotor stage of the disease [211, 212]. Consistent with theories about microglia activation as contributor to depression [213, 214], depressive symptoms may be considered as an early indicator of Parkinson’s disease. The protective effect that smoking exerts on PD could thus relate to α7 nAChR-mediated anti-inflammatory activity of nicotine [11]. This would imply that selective α7 nAChR agonists could be effective as prophylactic treatment for Parkinson’s disease. In this respect, it is worthwhile to mention that in MPTP-treated laboratory animals, the density of α7 nAChR increases (which is in contrast to α6β2-containing nAChRs, see above) [215]. The α7 nAChR agonist AQW051 [216] has been tested in patients with established Parkinson’s disease (ClinicalTrials.gov), but results remain to be published.

## Alzheimer’s disease

**Fig. 3:**
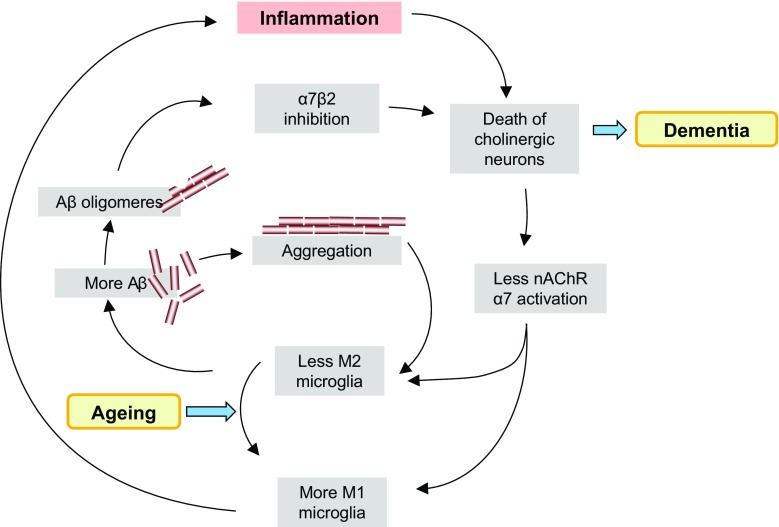
During aging the polarization of microglia gradually shifts towards the M1 phenotype. Diminution of M2 polarization presumably has negative consequences for Aβ catabolism. When extracellular Aβ levels increase, several positive feedback loops are triggered that ultimately lead to the demise of cholinergic neurons. Activation of α7 nAChRs counteracts the loss of the M2 phenotype. α7 nAChR agonists may furthermore compete with Aβ at (mitochondrial?) nicotinic α7β2 receptors. Treatment with α7 nAChR agonists might therefore delay the demise of cholinergic neurons, and thus delay the onset of dementia

One of the functions of microglia is to remove debris [217]. Microglia cells phagocytose and subsequently degrade Aβ, and thereby promote the removal of Aβ from the brain [218]. As mentioned before, microglia cells assume different phenotypes. With aging, microglia shift their morphology to a pro-inflammatory state and presumably lose their ability to phagocytose [219]. Activation of α7 nAChR expressed on microglia alters the phenotype and promotes phagocytosis and metabolism of Aβ [12]. Based on these data, one would expect that nicotine (smoking) and cholinesterase inhibitors would diminish Aβ load and improve Alzheimer disease. In transgenic animals, overexpressing Aβ beneficial effects of nicotine or ChE-I were indeed observed, while knock out of the α7 nAChR worsened pathology [12, 62, 220]. In patients with Alzheimer’s disease, cholinesterase inhibitors limit the cognitive deficits early in the course of the disease, but dosing and efficacy are limited by cholinergic (in particular muscarinic) side effects. For this reason, it is expected that α7 nAChR agonists might reach a similar therapeutic efficacy, but with less side effects. In a recent meta-analysis of prospective cohort studies investigating the effect of smoking on dementia and Alzheimer’s disease, smoking *increased* risk for dementia and AD [221]. This meta-analysis study included 37 studies with a total of almost 1 million patients and the statistical power was sufficient to investigate the influence of age. The increase in dementia risk was observed in the age group between 65 and 75, whereas the interpretation of results from patients older than 75 was hampered by a survival effect. Importantly, the increase in dementia or AD risk was not significant for smokers *under* 65 [221]. This result is clearly different from Parkinson’s disease, where smoking tendentially caused more benefit than harm [222]. Why would this be? The answer may lie in the special interaction of Aβ with α7 nAChRs. Parri et al. [223] have recently reviewed this in great detail. The fact that Aβ binds to α7 nAChRs has been observed and confirmed in numerous experimental settings, including post-mortem AD brain; however, it is unclear if this interaction results in inhibition or in stimulation of the receptor, and whether this interaction is reversible by agonists and antagonists. An intra-subunit allosteric binding pocket within the transmembrane domain of the α7 nAChR has been described as mechanism for non-competitive antagonism by Aβ (summarized by Parri et al. [223]). A further complication with relevance to AD pathology is the observation that cholinergic neurons in the basal forebrain express a heteromeric α7β2 isoform [224, 225]. Liu et al. [224] have reported that the α7β2 receptor is particularly sensitive to Aβ, since concentrations as low as 1 nM inhibited the functional responses to choline. They furthermore noted that inhibition was strongest with Aβ in its oligomeric form, followed by fibrillar Aβ, whereas monomeric Aβ was inactive. It is not clear why blockade of the α7β2 receptor is neurotoxic to cholinergic neurons, and if microglia cells from basal forebrain structures express this heteromeric nicotinic receptor. Nevertheless, one can begin to sketch a positive feedback process where aging causes polarization of microglia towards a phenotype that is less effective in phagocytosing and degrading Aβ. An overload of oligomeric Aβ in the extracellular space will block the α7β2 nicotinic receptor on basal forebrain cholinergic neurons, and these die as a consequence. Less acetylcholine leads to a reduction in α7 nAChR stimulation of microglia cells, which results in further loss of their phagocytic capacity. Diminished clearance of Aβ ultimately leads to extracellular precipitates, which presumably are a further trigger to microglia recruitment, inflammatory processes, and further toxicity to cholinergic neurons (see Fig. 3). In this process, an early intervention with nicotine, cholinesterase inhibitors, and in principle, α7 nAChR agonists may delay the start of the vicious circle. However, later in the process the availability of α7 nAChRs will be reduced by the negative interaction with the accumulating oligomeric Aβ. This would explain why the toxic effects of smoking become dominant over the neuroprotective effects in Alzheimer’s disease, but not (or less) in Parkinson’s disease. If the above-sketched vicious circle is correct, treatment with selective α7 nAChR agonists would be useful as prophylaxis, but less so for treatment of established severe AD-dementia.

## Discussion

From a historical perspective, selective α7 nAChR agonists have been targeted for cognitive deficits associated with schizophrenia (for recent reviews see [151, 152, 226]) and dementia in Alzheimer’s disease [227, 228]. Where investigated, the dose–response relationships for the pro-cognitive effects of α7 nAChR agonists usually display an inverted U-shape, indicating that higher doses are less effective than certain lower doses [142, 229–232]. Although the exact mechanism behind this profile remains speculative, it has been frequently noted that high concentrations of agonist cause receptor desensitization and suppression of functional responses [103, 233]. For instance, in in vitro experiments in oocytes, high concentrations of α7 nAChR agonists desensitized the receptor and blocked the calcium influx to the endogenous agonist acetylcholine [234]. Similar inhibitory effects on calcium influx were observed with synthetic α7 nAChR agonists at high concentrations [235]. Remarkably though, at lower concentrations a number of α7 nAChR agonists actually may potentiate the acetylcholine-induced response [236]. Estimations of brain levels at which α7 nAChR agonists evoke cognition-enhancing effects are in the range where they potentiate the acetylcholine-induced calcium influx in oocytes. It is therefore reasonable to assume that the pro-cognitive effect of α7 nAChR agonists is due to an enhancement of the acetylcholine-evoked response (known as the “co-agonist hypothesis,” see [236, 237]). This assumption is supported by preclinical data demonstrating an additive pro-cognitive effect of donepezil and the α7 nAChR agonist encenicline [236]. Although not yet reported, a similar co-agonist effect may occur at α7 receptors expressed by immune cells. If true, this would imply that low doses of a α7 nAChR agonist would be sufficient to enhance the anti-inflammatory response to the endogenous agonists (choline and/or acetylcholine).

In reviews dealing with potential indications of selective α7 nAChR agonists, the anti-inflammatory activity has never taken central stage (at least up to recently, see [64, 108]). The role that inflammation plays in the pathophysiology of psychiatric disorders is, however, well recognized [130, 140, 167, 171, 176, 238]. In the current review, we argue that several alternative indications for α7 agonists may be delineated from their effect on inflammation. Treatment with α7 nAChRs agonist may result in inhibition of the pro-inflammatory enzyme, GSK3β. In this respect, the treatment with α7-agonists resembles lithium treatment. This similarity may hold true not only for an indication like bipolar disorder (improvement in both manic and depressive symptoms) but also for suicide and neurological disorders. Suicide is a major cause of death in depression, bipolar disorder, and schizophrenia. Clinical data have accumulated which indicate that inflammation and microglia M1-polarization contribute to the pathophysiology of suicide *independent* from the underlying psychiatric disease [239–242]. Diverse pro-inflammatory mechanisms such as autoimmunity, neurotropic pathogens, stress, or traumatic brain injury have been documented in suicidal patients [243]. Since α7 nAChR stimulation in immune cells can result in GSK3β inhibition, and since the GSK3-inhibitor, lithium [244], is a recognized anti-inflammatory [245] *and* anti-suicidal compound [246, 247], one may propose the use of nicotine α7-agonists for prevention of suicide. Chronic treatment with lithium by virtue of its GSK3β inhibitory effect also ameliorated the disease processes in preclinical models of multiple sclerosis [248], Alzheimer [249–251], and Parkinson’s disease [252–254]. It should be noted though that GSK3β inhibition by lithium in these models is just a symptomatic treatment and does not stop the underlying pathological processes. Consequently, once the pharmacotherapy is interrupted, the disease is likely to return. Nevertheless, treatment with a α7 nAChR agonist might constitute a safe alternative to lithium and one could propose the use of α7 nAChR agonists for these neurological disorders.

In contrast to lithium treatment, the beneficial effect of α7 nAChR stimulation is lost when the receptor is defective (or missing all together). An example was presented in the autism section, and also in late-stage Alzheimer’s disease, the nicotine receptor owing to the interaction with the β-amyloid protein may become severely dysfunctional. One potential explanation for the lack of robust effects of α7 nAChR agonists on cognitive function in schizophrenia is the rapid desensitization of α7 nAChRs. This is a general concern and applies to any α7 nAChR agonist indication. A simple biomarker test for quantification of α7 nAChR stimulation on an inflammation read-out would thus be highly desirable. Perhaps the receptor desensitization of α7 nAChRs may be less of an issue for inflammation-related indications. In this context, it is remarkable that basically all α7 agonists were effective against negative symptoms of schizophrenia (while effects on cognition were equivocal). We would also expect that the beneficial effects of smoking and cholinesterase-inhibitor-treatment would be absent if α7 nAChRs would be desensitized for most of the time. However, it must be admitted that a thorough investigation of the concentration/response relationship for the anti-inflammatory effect of any of the selective α7 nAChR agonists remains to be determined.

Since epidemiological data suggest that smoking exerts a protective effect in Parkinson’s disease, α7 nAChR agonists might be a preferable alternative to cholinesterase inhibition and smoking. Registration studies for prophylactic indications such as in Parkinson’s or Alzheimer’s disease are however difficult, long lasting, and expensive, and it is unlikely that companies will invest in these indications without the opportunity for registration in an acute disorder. What could be the pioneer indication for α7 nAChR agonists? Based on clinical information from vagus nerve stimulation treatment, depression would be a logical choice. Unfortunately, depression studies generally suffer from a high placebo response, making also the indication ‘depression’ less attractive.

We currently favor l-DOPA-induced dyskinesia as possible ‘pioneer indication.’ It has been published that two chemically distinct α7 nAChR agonists, ABT-107 and AQW051, suppress l-DOPA-induced dyskinesias in (MPTP-treated) Parkinsonian monkeys [255, 256]. The precise mechanism by which this response is brought about remains unknown; however, again it may involve GSK3 inhibition, since low-dose lithium was also recently shown to be active in a similar Parkinson model in mice [254]. The beneficial effect of α7 nAChR agonists may involve attenuation of MPTP-induced neuroinflammation and protection dopamine neurons in the substantia nigra pars compacta [206]. Thus, efficacy for α7 nAChR agonists in clinical trials of l-DOPA-induced dyskinesias could serve as a clinical entry point to pave the way for indications where longer treatment regimens are warranted.
